# Supplementary figures and images for: Regulation of immune receptor kinase plasma membrane nanoscale organization by a plant peptide hormone and its receptors (part 1 of 2)
Source: eLife. 2022 Jan 6;11:e74162. doi: 10.7554/eLife.74162 (PMC8791635; doi:10.7554/eLife.74162)

Source Data Fig. 2A

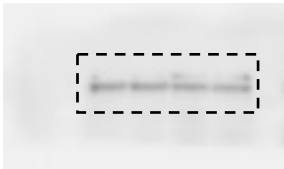

$\alpha$ -FLS2 input

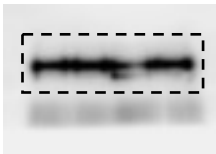

$\alpha$ -FLS2 IP

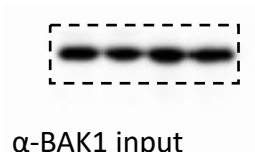

$\alpha$ -BAK1 input

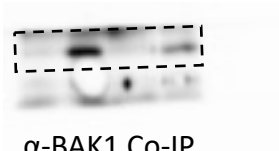

$\alpha$ -BAK1 Co-IP

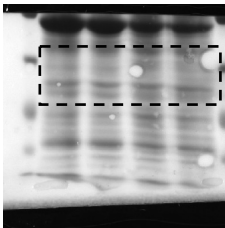

CBB input

Supplement: Figure 2—source data 2. [file elife-74162-fig2-data2.pdf]

Source Data Figure 2 – supplement figure 2

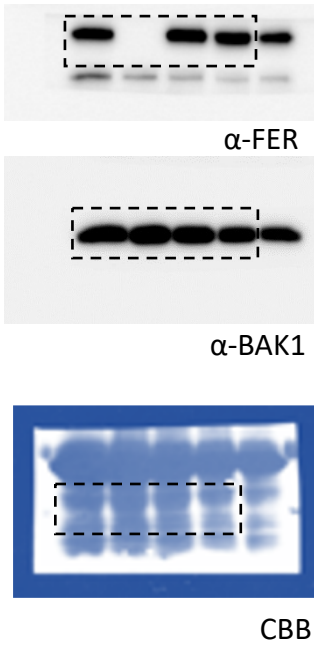

Supplement: Figure 2—figure supplement 2—source data 1. [file elife-74162-fig2-figsupp2-data1.pdf]

Source Data Figure 3 – supplement figure 1

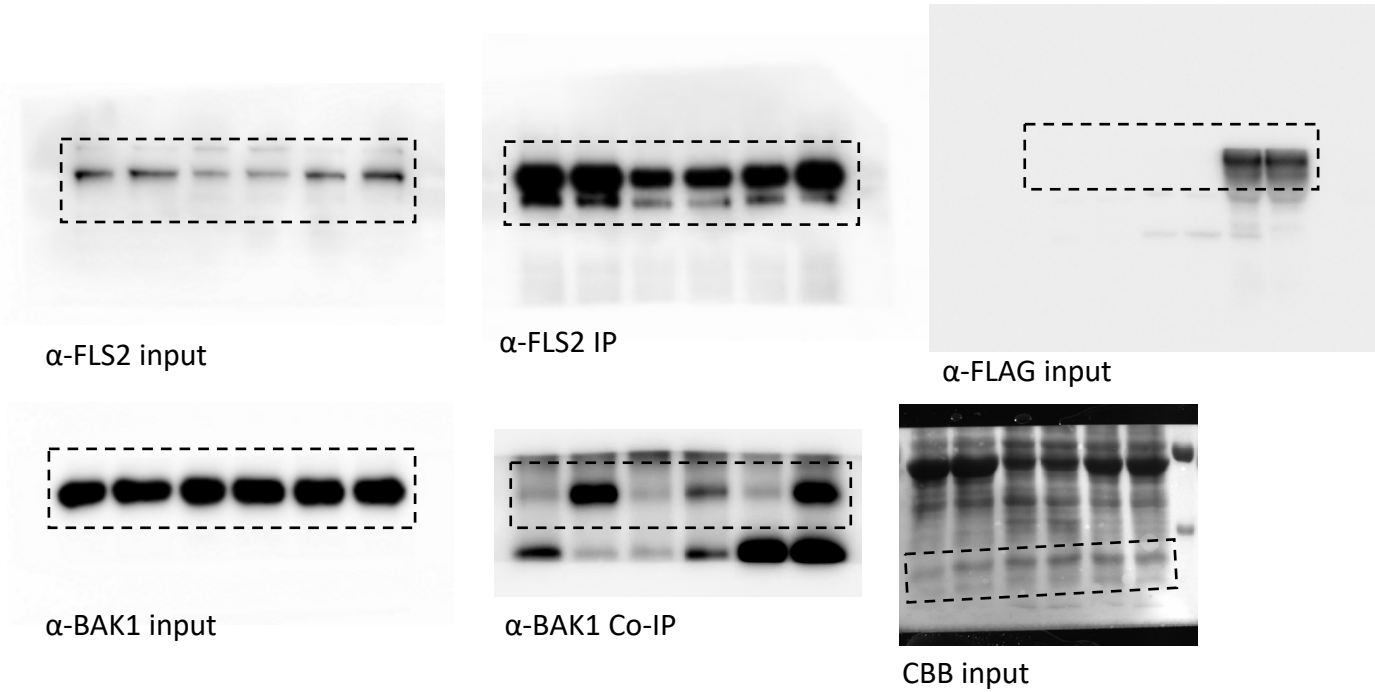

Supplement: Figure 3—figure supplement 1—source data 2. [file elife-74162-fig3-figsupp1-data2.pdf]

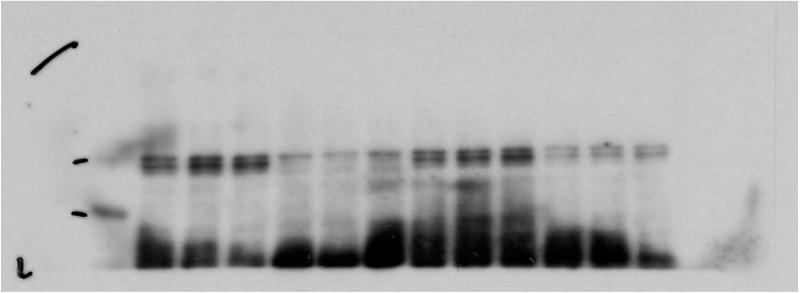

Supplement: Source data 1. [file elife-74162-supp1.zip › Figure 4 - supplement figure 8I/Input_FLS2.png]

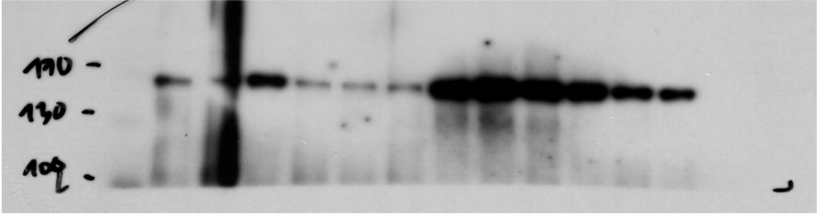

Supplement: Source data 1. [file elife-74162-supp1.zip › Figure 4 - supplement figure 8I/IP_FLS2.png]

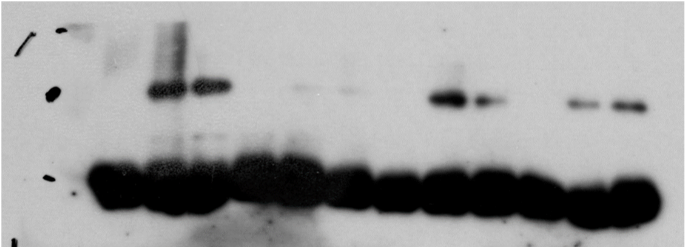

Supplement: Source data 1. [file elife-74162-supp1.zip › Figure 4 - supplement figure 8I/IP_BAK1.png]

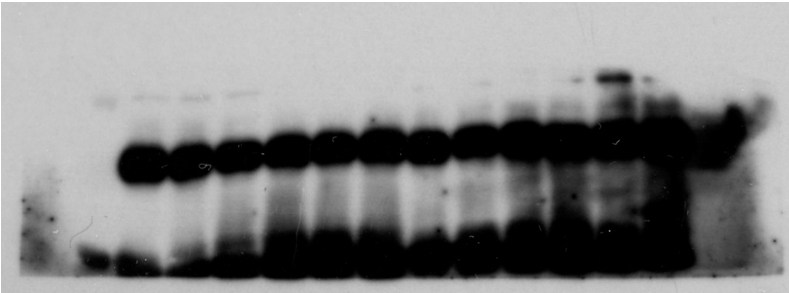

Supplement: Source data 1. [file elife-74162-supp1.zip › Figure 4 - supplement figure 8I/Input_BAK1.png]

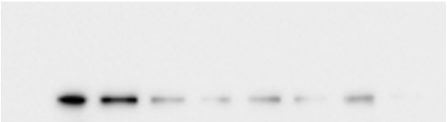

Supplement: Source data 1. [file elife-74162-supp1.zip › Figure 2 - supplement figure 1 - source data blots images/B_FLS2IP.png]

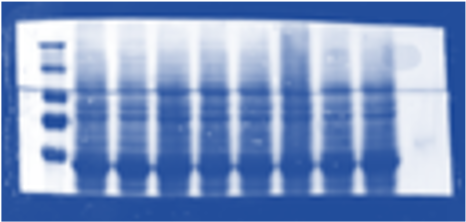

Supplement: Source data 1. [file elife-74162-supp1.zip › Figure 2 - supplement figure 1 - source data blots images/B_CBB.png]

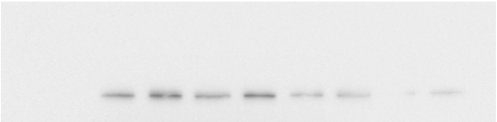

Supplement: Source data 1. [file elife-74162-supp1.zip › Figure 2 - supplement figure 1 - source data blots images/B_FLS2input.png]

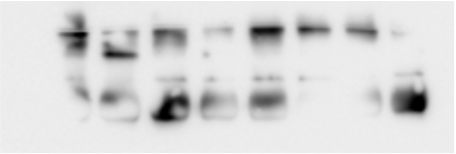

Supplement: Source data 1. [file elife-74162-supp1.zip › Figure 2 - supplement figure 1 - source data blots images/B_BAK1IP.png]

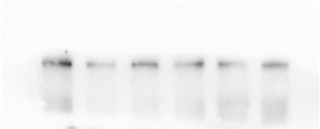

Supplement: Source data 1. [file elife-74162-supp1.zip › Figure 2 - supplement figure 1 - source data blots images/A_FLS2_IP.png]

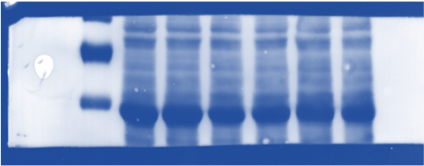

Supplement: Source data 1. [file elife-74162-supp1.zip › Figure 2 - supplement figure 1 - source data blots images/A_CBB.png]

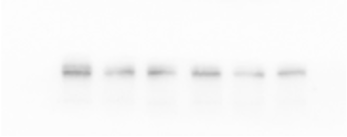

Supplement: Source data 1. [file elife-74162-supp1.zip › Figure 2 - supplement figure 1 - source data blots images/A_FLS2input.png]

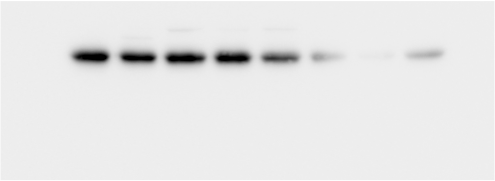

Supplement: Source data 1. [file elife-74162-supp1.zip › Figure 2 - supplement figure 1 - source data blots images/B_BAK1input.png]

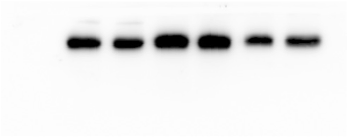

Supplement: Source data 1. [file elife-74162-supp1.zip › Figure 2 - supplement figure 1 - source data blots images/A_BAK1_input.png]

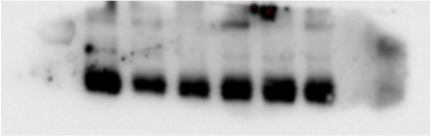

Supplement: Source data 1. [file elife-74162-supp1.zip › Figure 2 - supplement figure 1 - source data blots images/A_BAK1_IP.png]

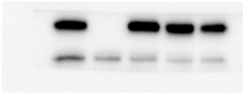

Supplement: Source data 1. [file elife-74162-supp1.zip › Figure 2 - supplement figure 2 - source data blots images/FER.png]

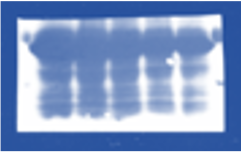

Supplement: Source data 1. [file elife-74162-supp1.zip › Figure 2 - supplement figure 2 - source data blots images/CBB.png]

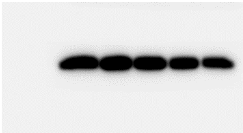

Supplement: Source data 1. [file elife-74162-supp1.zip › Figure 2 - supplement figure 2 - source data blots images/BAK1.png]

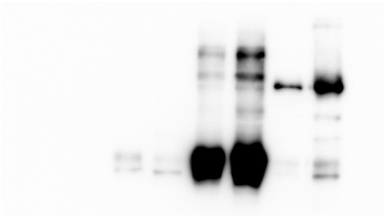

Supplement: Source data 1. [file elife-74162-supp1.zip › Figure 2 - supplement figure 5 - source data blots images/a-FLAG_input.png]

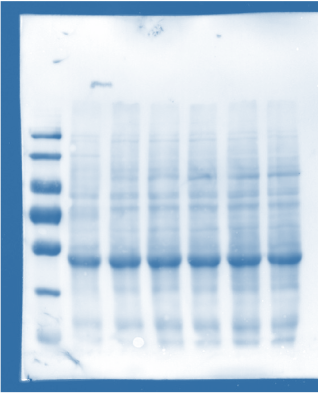

Supplement: Source data 1. [file elife-74162-supp1.zip › Figure 2 - supplement figure 5 - source data blots images/CBB.png]

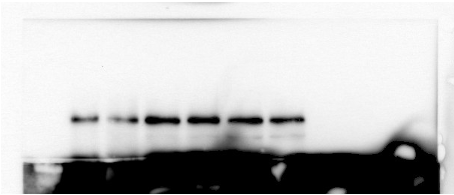

Supplement: Source data 1. [file elife-74162-supp1.zip › Figure 2 - supplement figure 5 - source data blots images/a-FER_input.png]

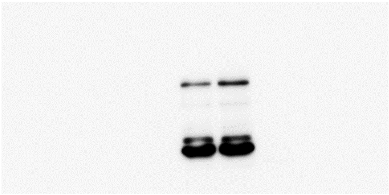

Supplement: Source data 1. [file elife-74162-supp1.zip › Figure 2 - supplement figure 5 - source data blots images/a-FER_CoIP.png]

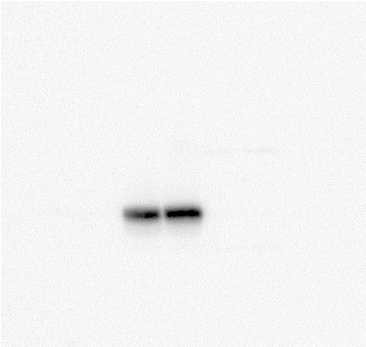

Supplement: Source data 1. [file elife-74162-supp1.zip › Figure 2 - supplement figure 5 - source data blots images/a-FLAG_IP.png]

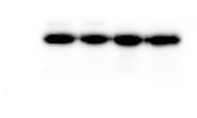

Supplement: Source data 1. [file elife-74162-supp1.zip › Figure 2A - source data blots images/a-BAK1_input.png]

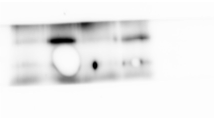

Supplement: Source data 1. [file elife-74162-supp1.zip › Figure 2A - source data blots images/a-BAK1_IP.png]

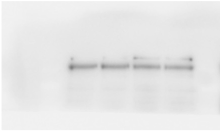

Supplement: Source data 1. [file elife-74162-supp1.zip › Figure 2A - source data blots images/a-FLS2_input.png]

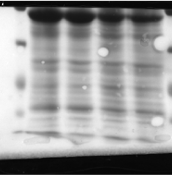

Supplement: Source data 1. [file elife-74162-supp1.zip › Figure 2A - source data blots images/CBB.png]

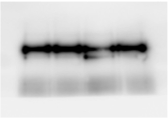

Supplement: Source data 1. [file elife-74162-supp1.zip › Figure 2A - source data blots images/a-FLS2_IP.png]

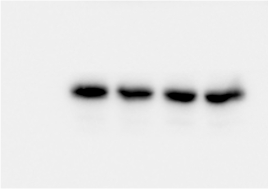

Supplement: Source data 1. [file elife-74162-supp1.zip › Figure 3 - supplement figure 1 - source data blots images/replicate2/BAK1input.png]

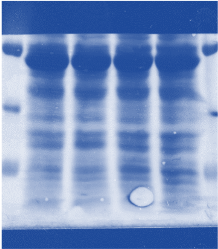

Supplement: Source data 1. [file elife-74162-supp1.zip › Figure 3 - supplement figure 1 - source data blots images/replicate2/CBB.png]

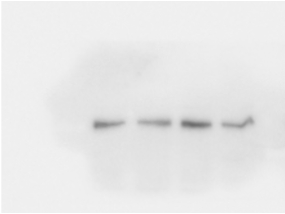

Supplement: Source data 1. [file elife-74162-supp1.zip › Figure 3 - supplement figure 1 - source data blots images/replicate2/FLS2input.png]

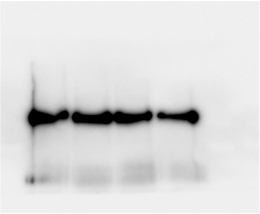

Supplement: Source data 1. [file elife-74162-supp1.zip › Figure 3 - supplement figure 1 - source data blots images/replicate2/FLS2IP.png]

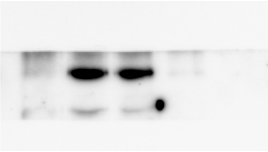

Supplement: Source data 1. [file elife-74162-supp1.zip › Figure 3 - supplement figure 1 - source data blots images/replicate2/BAK1IP.png]

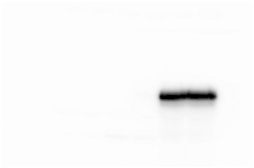

Supplement: Source data 1. [file elife-74162-supp1.zip › Figure 3 - supplement figure 1 - source data blots images/replicate3/FLAGinput.png]

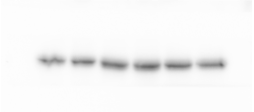

Supplement: Source data 1. [file elife-74162-supp1.zip › Figure 3 - supplement figure 1 - source data blots images/replicate3/BAK1input.png]

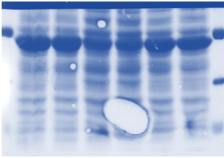

Supplement: Source data 1. [file elife-74162-supp1.zip › Figure 3 - supplement figure 1 - source data blots images/replicate3/CBB.png]

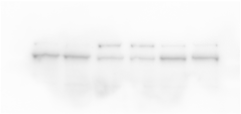

Supplement: Source data 1. [file elife-74162-supp1.zip › Figure 3 - supplement figure 1 - source data blots images/replicate3/FLS2input.png]

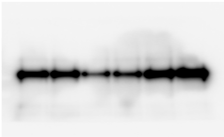

Supplement: Source data 1. [file elife-74162-supp1.zip › Figure 3 - supplement figure 1 - source data blots images/replicate3/FLS2IP.png]

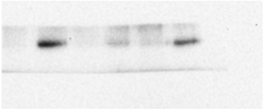

Supplement: Source data 1. [file elife-74162-supp1.zip › Figure 3 - supplement figure 1 - source data blots images/replicate3/BAK1IP.png]

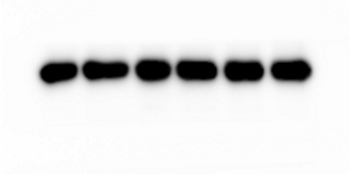

Supplement: Source data 1. [file elife-74162-supp1.zip › Figure 3 - supplement figure 1 - source data blots images/replicate1/a-BAK1_input.png]

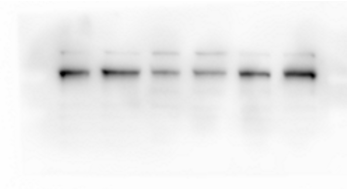

Supplement: Source data 1. [file elife-74162-supp1.zip › Figure 3 - supplement figure 1 - source data blots images/replicate1/a-FLS2_input.png]

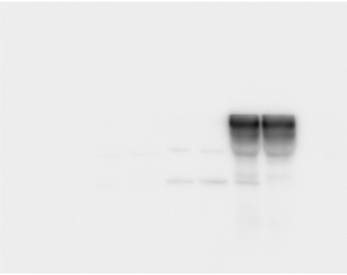

Supplement: Source data 1. [file elife-74162-supp1.zip › Figure 3 - supplement figure 1 - source data blots images/replicate1/a-FLAG_input.png]

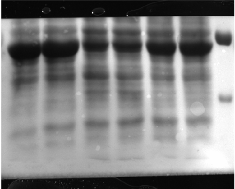

Supplement: Source data 1. [file elife-74162-supp1.zip › Figure 3 - supplement figure 1 - source data blots images/replicate1/CBB.png]

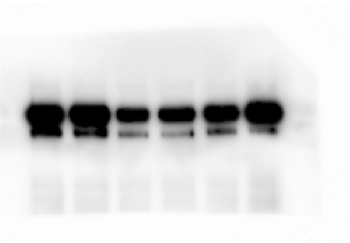

Supplement: Source data 1. [file elife-74162-supp1.zip › Figure 3 - supplement figure 1 - source data blots images/replicate1/a-FLS2_IP.png]

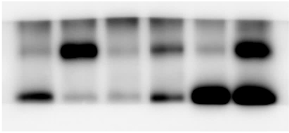

Supplement: Source data 1. [file elife-74162-supp1.zip › Figure 3 - supplement figure 1 - source data blots images/replicate1/a-BAK1_CoIP.png]

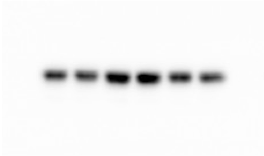

Supplement: Source data 1. [file elife-74162-supp1.zip › Figure 3E - source data blot images/a-BAK1_input.png]

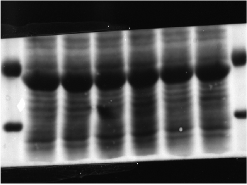

Supplement: Source data 1. [file elife-74162-supp1.zip › Figure 3E - source data blot images/CBB_input.png]

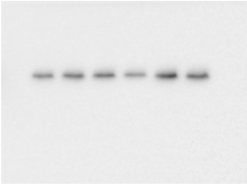

Supplement: Source data 1. [file elife-74162-supp1.zip › Figure 3E - source data blot images/a-FLS2_input.png]

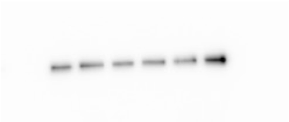

Supplement: Source data 1. [file elife-74162-supp1.zip › Figure 3E - source data blot images/a-FLS2_IP.png]

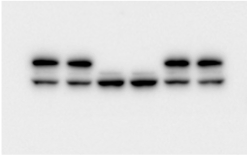

Supplement: Source data 1. [file elife-74162-supp1.zip › Figure 3E - source data blot images/a-FER_input.png]

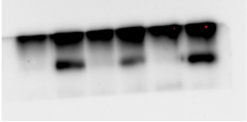

Supplement: Source data 1. [file elife-74162-supp1.zip › Figure 3E - source data blot images/a-BAK1_CoIP.png]

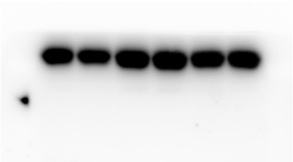

Supplement: Source data 1. [file elife-74162-supp1.zip › Figure 4 - supplement figure 1 - source data blots images/a-BAK1_input.png]

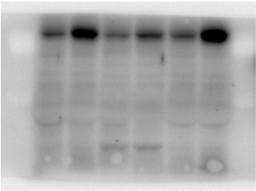

Supplement: Source data 1. [file elife-74162-supp1.zip › Figure 4 - supplement figure 1 - source data blots images/a-BAK1_S612_input.png]

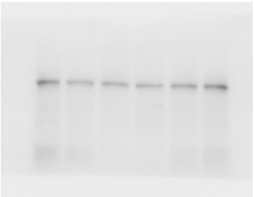

Supplement: Source data 1. [file elife-74162-supp1.zip › Figure 4 - supplement figure 1 - source data blots images/a-FLS2_input.png]

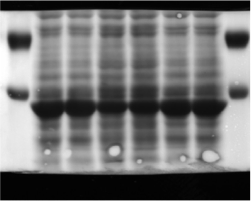

Supplement: Source data 1. [file elife-74162-supp1.zip › Figure 4 - supplement figure 1 - source data blots images/CBB.png]

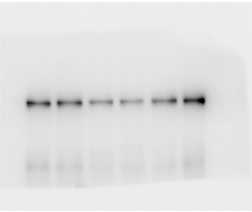

Supplement: Source data 1. [file elife-74162-supp1.zip › Figure 4 - supplement figure 1 - source data blots images/a-FLS2_IP.png]

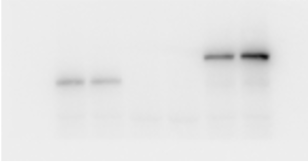

Supplement: Source data 1. [file elife-74162-supp1.zip › Figure 4 - supplement figure 1 - source data blots images/a-FER_input.png]

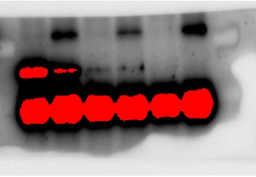

Supplement: Source data 1. [file elife-74162-supp1.zip › Figure 4 - supplement figure 1 - source data blots images/a-BAK1_CoIP.png]

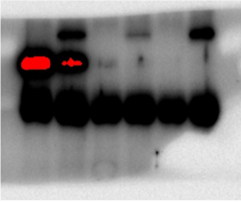

Supplement: Source data 1. [file elife-74162-supp1.zip › Figure 4 - supplement figure 1 - source data blots images/a-BAK1_S612_CoIP.png]

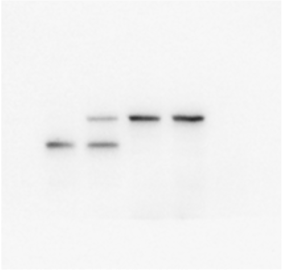

Supplement: Source data 1. [file elife-74162-supp1.zip › Figure 4 - supplement figure 2A - source datab blots images/a-FER.png]

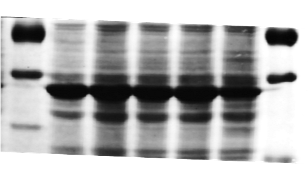

Supplement: Source data 1. [file elife-74162-supp1.zip › Figure 4 - supplement figure 2A - source datab blots images/CBB.png]

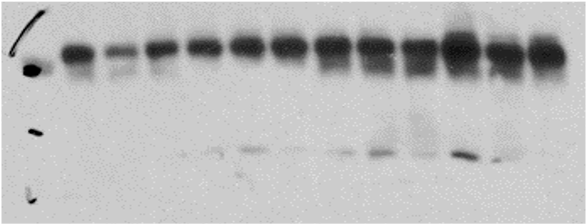

Supplement: Source data 1. [file elife-74162-supp1.zip › Figure 4 - supplement figure 2B - source data blots images/Replicate2/BAK1Input.png]

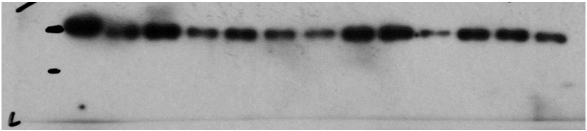

Supplement: Source data 1. [file elife-74162-supp1.zip › Figure 4 - supplement figure 2B - source data blots images/Replicate2/IPFLS2.png]

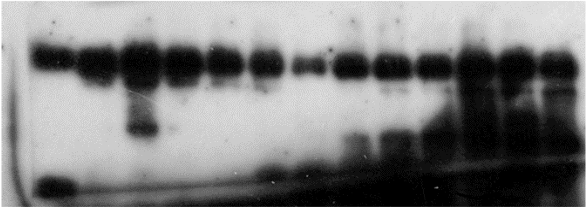

Supplement: Source data 1. [file elife-74162-supp1.zip › Figure 4 - supplement figure 2B - source data blots images/Replicate2/FLS2Input.png]

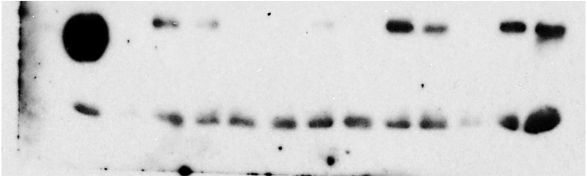

Supplement: Source data 1. [file elife-74162-supp1.zip › Figure 4 - supplement figure 2B - source data blots images/Replicate2/CoIPBAK1.png]

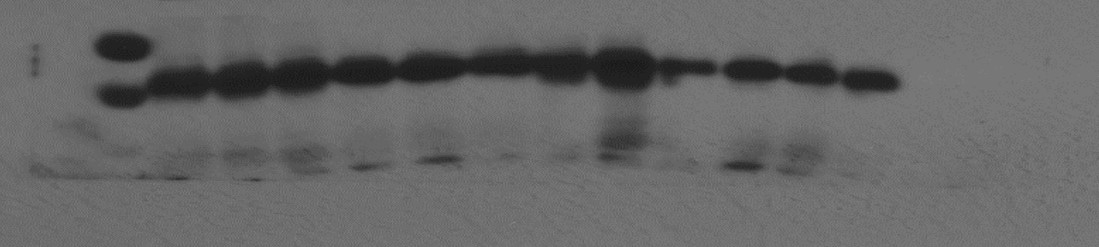

Supplement: Source data 1. [file elife-74162-supp1.zip › Figure 4 - supplement figure 2B - source data blots images/Replicate1/Input a-BAK1.jpg]

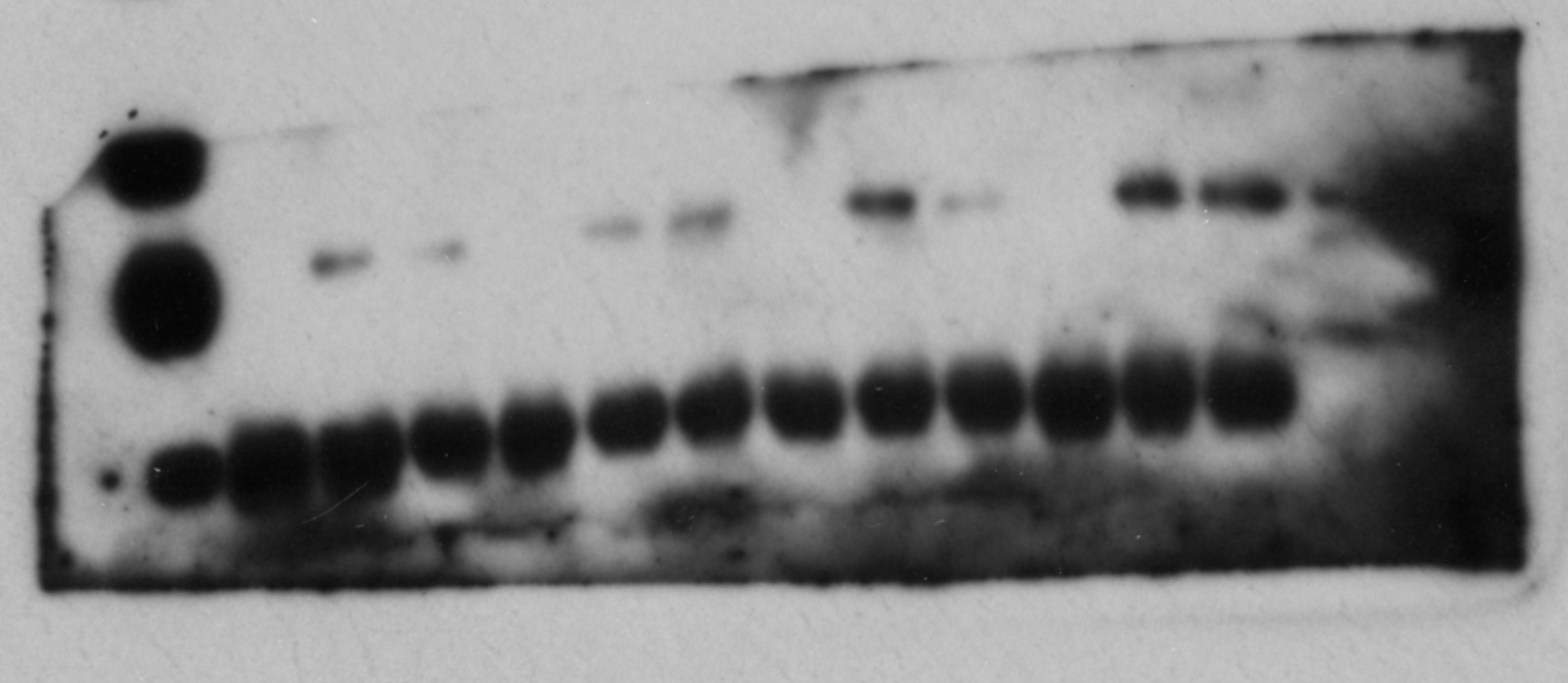

Supplement: Source data 1. [file elife-74162-supp1.zip › Figure 4 - supplement figure 2B - source data blots images/Replicate1/IP a-BAK1.jpg]

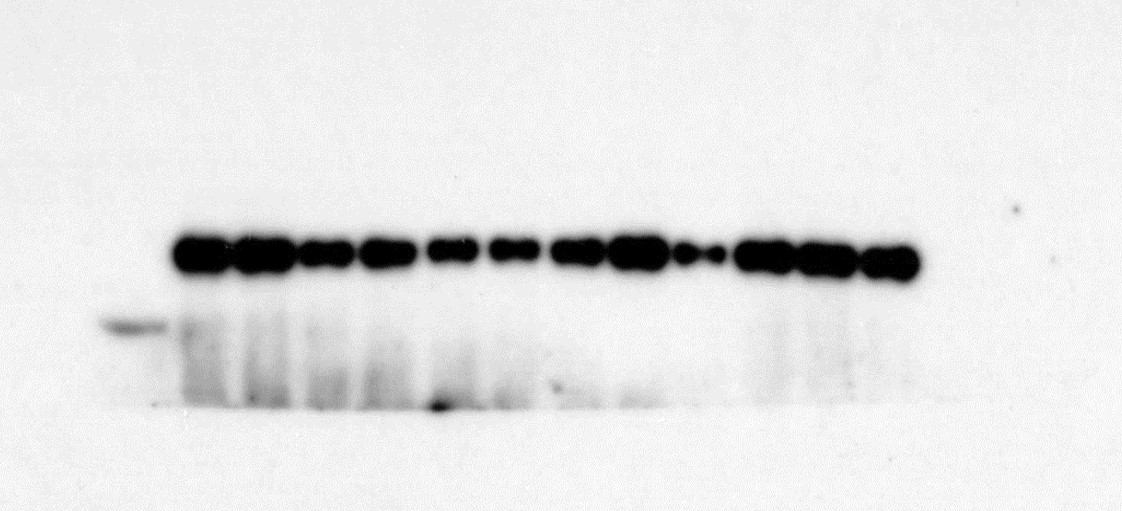

Supplement: Source data 1. [file elife-74162-supp1.zip › Figure 4 - supplement figure 2B - source data blots images/Replicate1/IP a-FLS2.jpg]

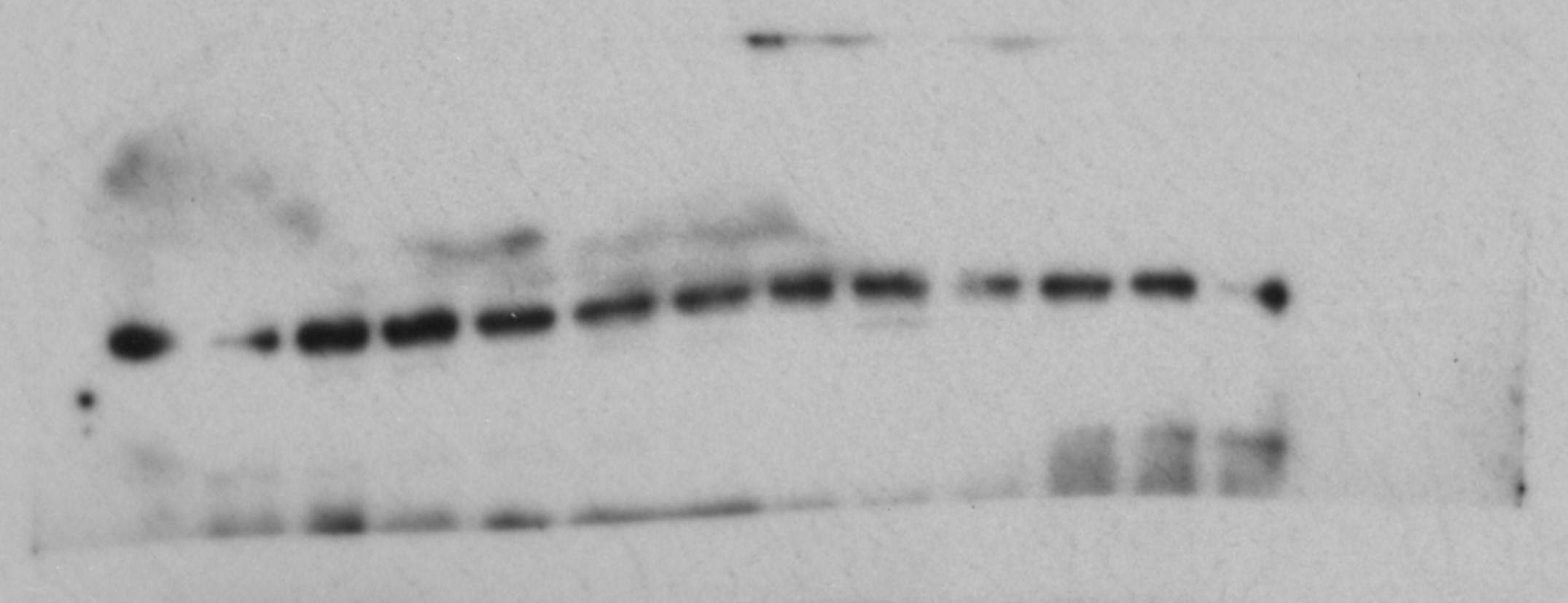

Supplement: Source data 1. [file elife-74162-supp1.zip › Figure 4 - supplement figure 2B - source data blots images/Replicate1/Input a-FLS2.jpg]

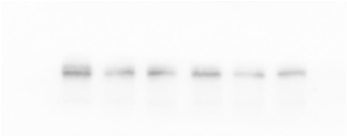

Supplement: Source data 1. [file elife-74162-supp1.zip › Figure 4 - supplement figure 8A/Input_FLS2.png]

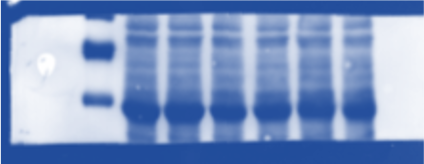

Supplement: Source data 1. [file elife-74162-supp1.zip › Figure 4 - supplement figure 8A/CBB.png]

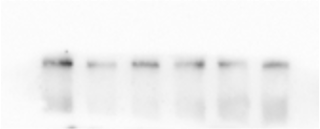

Supplement: Source data 1. [file elife-74162-supp1.zip › Figure 4 - supplement figure 8A/IP_FLS2.png]

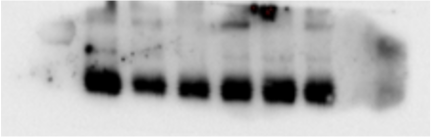

Supplement: Source data 1. [file elife-74162-supp1.zip › Figure 4 - supplement figure 8A/IP_BAK1.png]

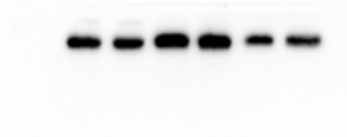

Supplement: Source data 1. [file elife-74162-supp1.zip › Figure 4 - supplement figure 8A/Input_BAK1.png]

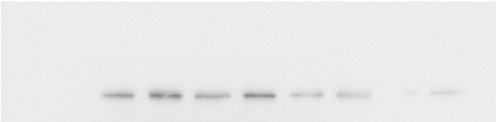

Supplement: Source data 1. [file elife-74162-supp1.zip › Figure 4 - supplement figure 8B/Input_FLS2.png]

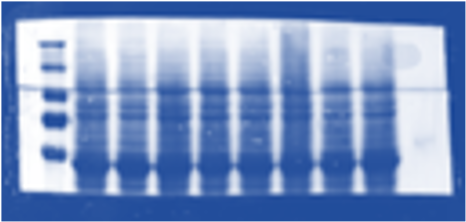

Supplement: Source data 1. [file elife-74162-supp1.zip › Figure 4 - supplement figure 8B/CBB.png]

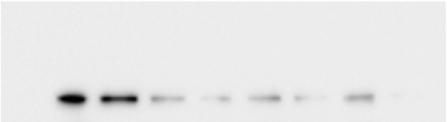

Supplement: Source data 1. [file elife-74162-supp1.zip › Figure 4 - supplement figure 8B/IP_FLS2.png]

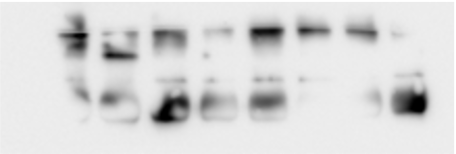

Supplement: Source data 1. [file elife-74162-supp1.zip › Figure 4 - supplement figure 8B/IP_BAK1.png]

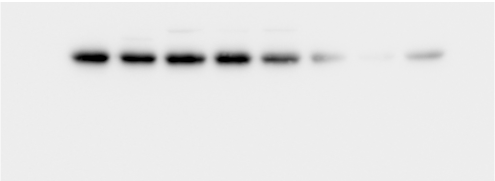

Supplement: Source data 1. [file elife-74162-supp1.zip › Figure 4 - supplement figure 8B/Input_BAK1.png]

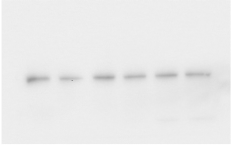

Supplement: Source data 1. [file elife-74162-supp1.zip › Figure 4 - supplement figure 8C/Input_FLS2.png]

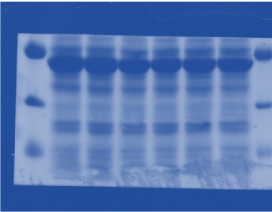

Supplement: Source data 1. [file elife-74162-supp1.zip › Figure 4 - supplement figure 8C/CBB.png]

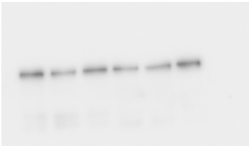

Supplement: Source data 1. [file elife-74162-supp1.zip › Figure 4 - supplement figure 8C/IP_FLS2.png]

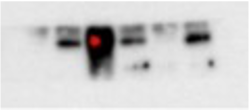

Supplement: Source data 1. [file elife-74162-supp1.zip › Figure 4 - supplement figure 8C/IP_BAK1.png]

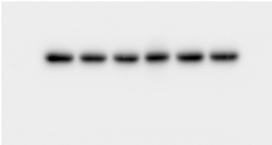

Supplement: Source data 1. [file elife-74162-supp1.zip › Figure 4 - supplement figure 8C/Input_BAK1.png]

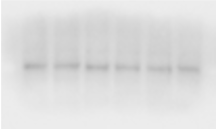

Supplement: Source data 1. [file elife-74162-supp1.zip › Figure 4 - supplement figure 8D/Input_FLS2.png]

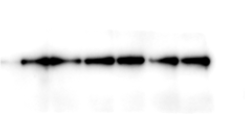

Supplement: Source data 1. [file elife-74162-supp1.zip › Figure 4 - supplement figure 8D/IP_FLS2.png]

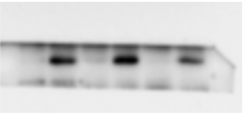

Supplement: Source data 1. [file elife-74162-supp1.zip › Figure 4 - supplement figure 8D/IP_BAK1.png]

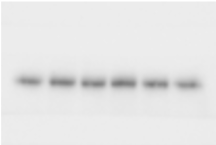

Supplement: Source data 1. [file elife-74162-supp1.zip › Figure 4 - supplement figure 8D/Input_BAK1.png]

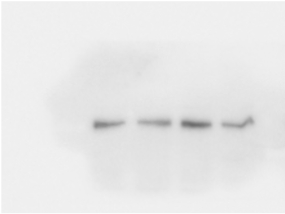

Supplement: Source data 1. [file elife-74162-supp1.zip › Figure 4 - supplement figure 8E/Input_FLS2.png]

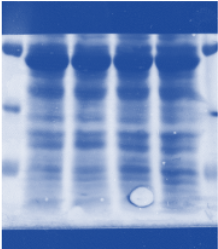

Supplement: Source data 1. [file elife-74162-supp1.zip › Figure 4 - supplement figure 8E/CBB.png]

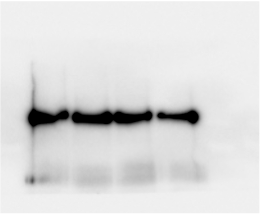

Supplement: Source data 1. [file elife-74162-supp1.zip › Figure 4 - supplement figure 8E/IP_FLS2.png]

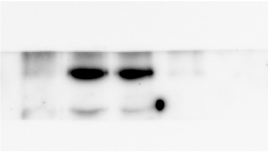

Supplement: Source data 1. [file elife-74162-supp1.zip › Figure 4 - supplement figure 8E/IP_BAK1.png]

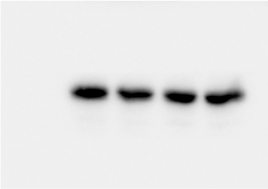

Supplement: Source data 1. [file elife-74162-supp1.zip › Figure 4 - supplement figure 8E/Input_BAK1.png]

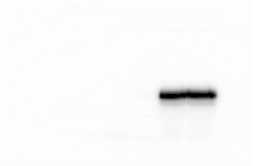

Supplement: Source data 1. [file elife-74162-supp1.zip › Figure 4 - supplement figure 8F/Input_FLAG.png]

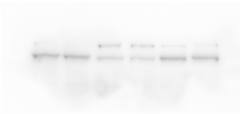

Supplement: Source data 1. [file elife-74162-supp1.zip › Figure 4 - supplement figure 8F/Input_FLS2.png]

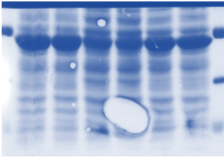

Supplement: Source data 1. [file elife-74162-supp1.zip › Figure 4 - supplement figure 8F/CBB.png]

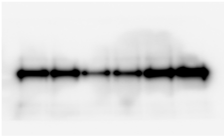

Supplement: Source data 1. [file elife-74162-supp1.zip › Figure 4 - supplement figure 8F/IP_FLS2.png]

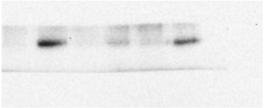

Supplement: Source data 1. [file elife-74162-supp1.zip › Figure 4 - supplement figure 8F/IP_BAK1.png]
