# Supplementary material for: Regulation of immune receptor kinase plasma membrane nanoscale organization by a plant peptide hormone and its receptors
Source: eLife. 2022 Jan 6;11:e74162. doi: 10.7554/eLife.74162 (PMC8791635; doi:10.7554/eLife.74162)
Supplement: Figure 2—figure supplement 5—source data 1. [file elife-74162-fig2-figsupp5-data1.pdf]

Source Data Figure 2 – supplement figure 5

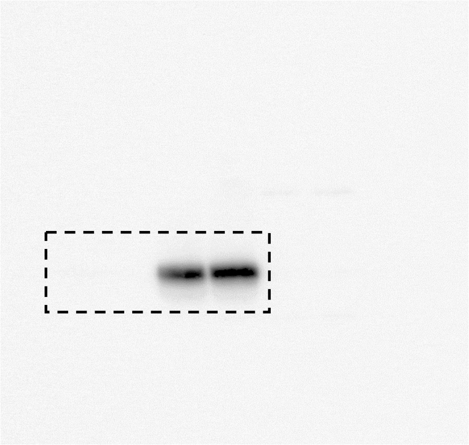

α-FLAG IP

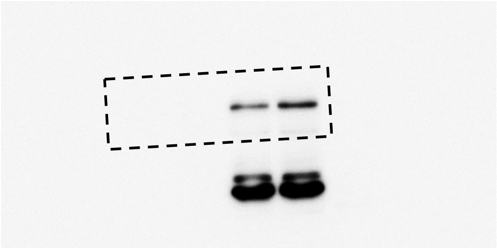

α-FER Co-IP

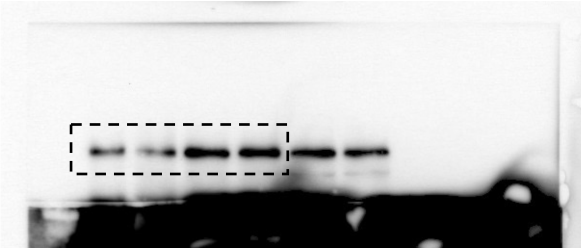

α-FER input

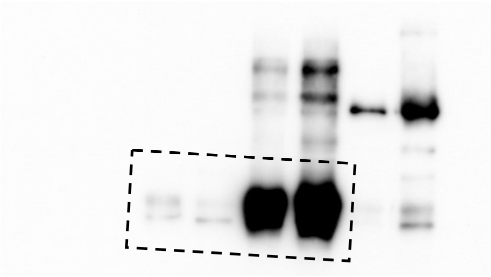

α-FLAG input

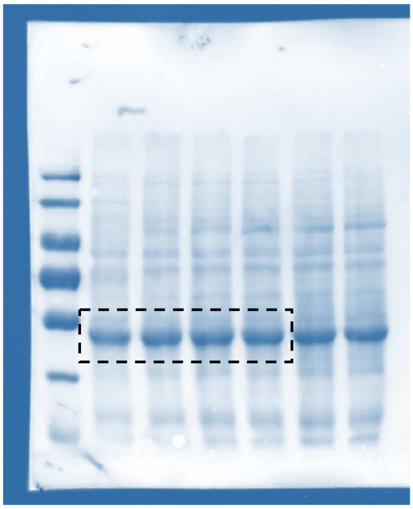

CBB
