# Supplementary material for: Regulation of immune receptor kinase plasma membrane nanoscale organization by a plant peptide hormone and its receptors
Source: eLife. 2022 Jan 6;11:e74162. doi: 10.7554/eLife.74162 (PMC8791635; doi:10.7554/eLife.74162)
Supplement: Figure 3—source data 2. [file elife-74162-fig3-data2.pdf]

Source Data Fig. 3E

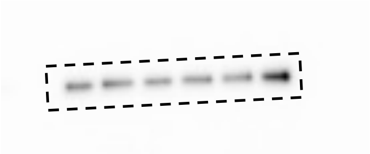

α-FLS2 IP

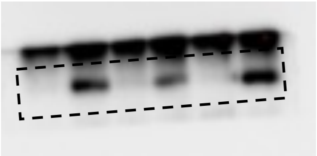

α-BAK1 Co-IP

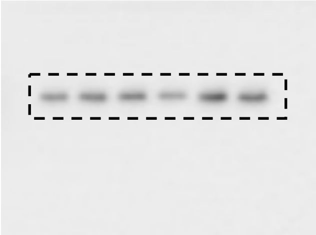

α-FLS2 input

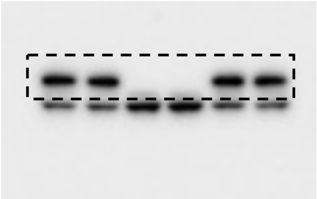

α-FER input

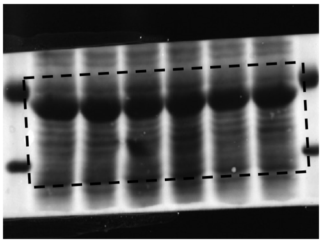

CBB input

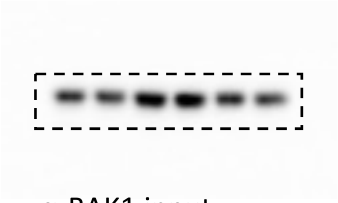

α-BAK1 input
