# Supplementary material for: Regulation of immune receptor kinase plasma membrane nanoscale organization by a plant peptide hormone and its receptors
Source: eLife. 2022 Jan 6;11:e74162. doi: 10.7554/eLife.74162 (PMC8791635; doi:10.7554/eLife.74162)
Supplement: Figure 4—figure supplement 8—source data 1. [file elife-74162-fig4-figsupp8-data1.pdf]

Source Data Figure 4 – supplement figure 8A

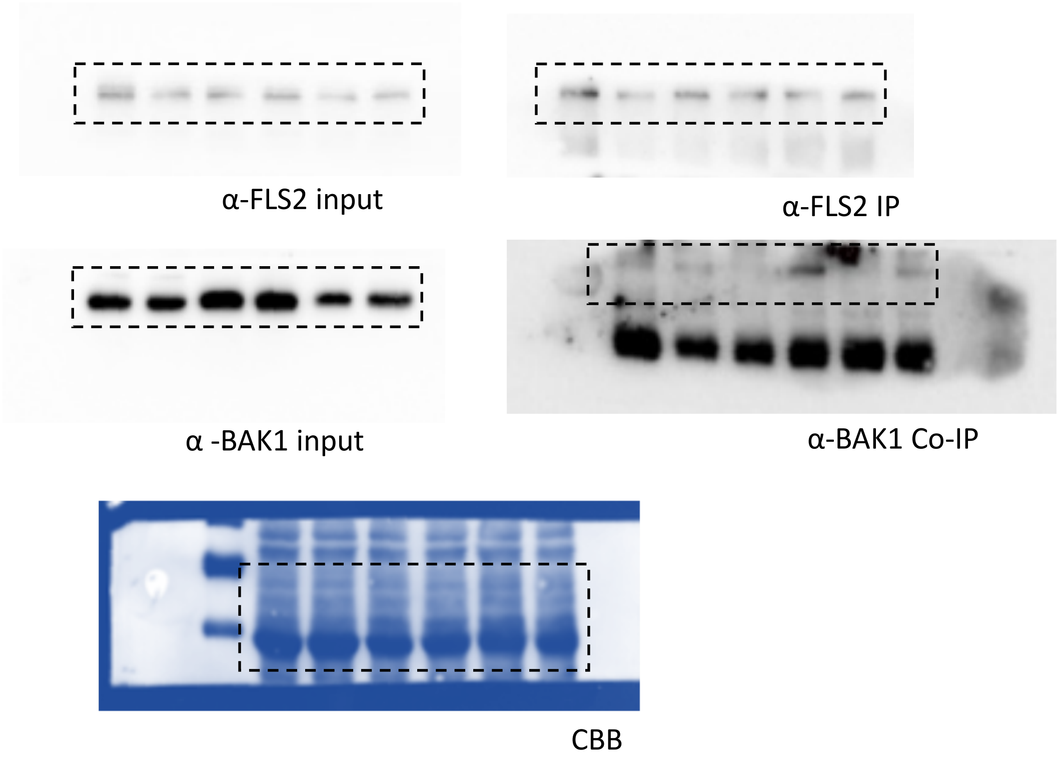

Source Data Figure 4 – supplement figure 8B

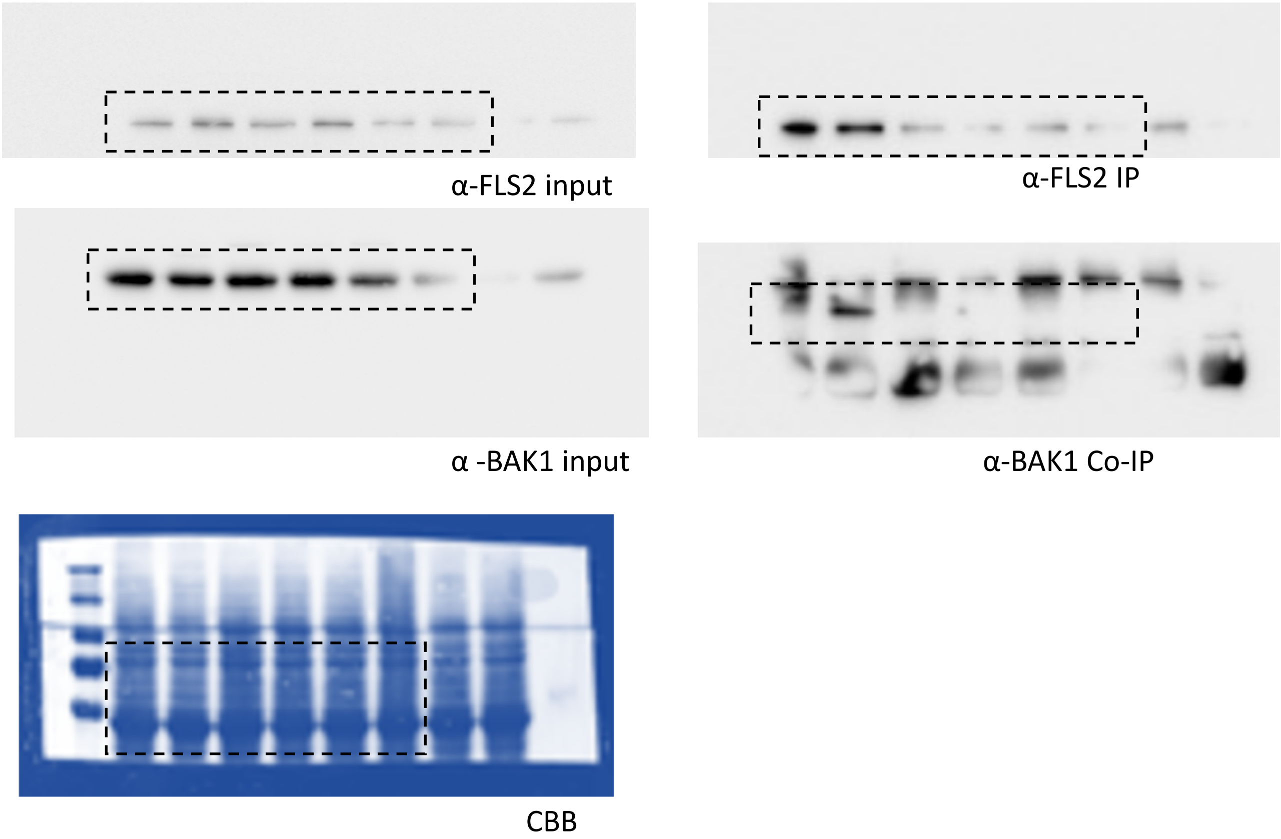

Source Data Figure 4 – supplement figure 8E

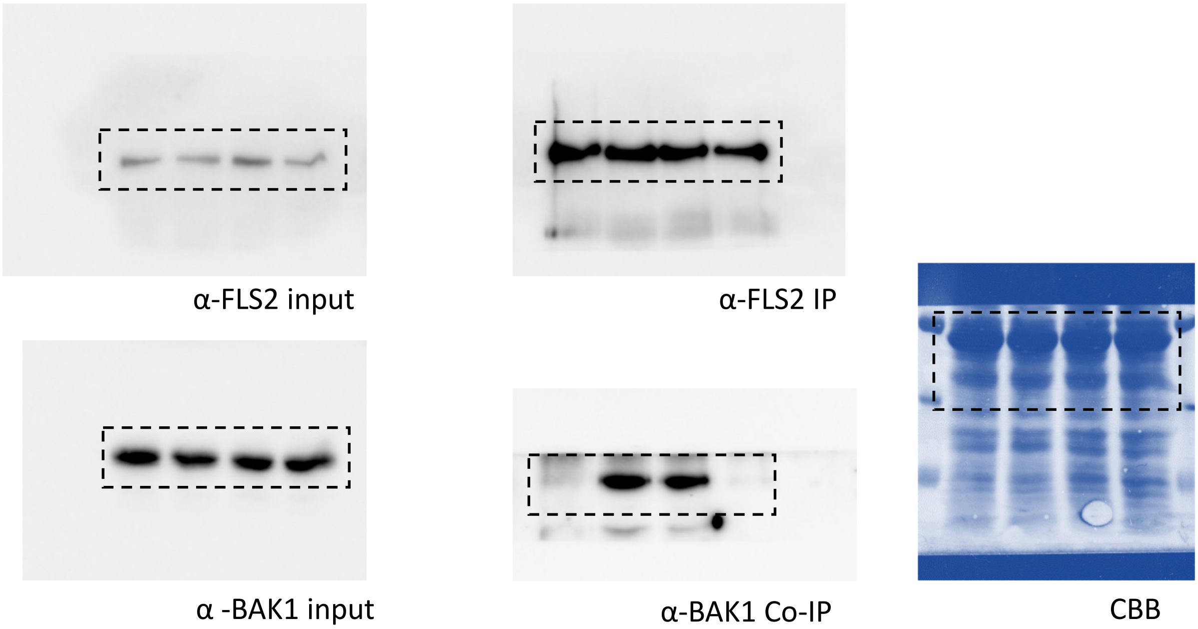

Source Data Figure 4 – supplement figure 8F

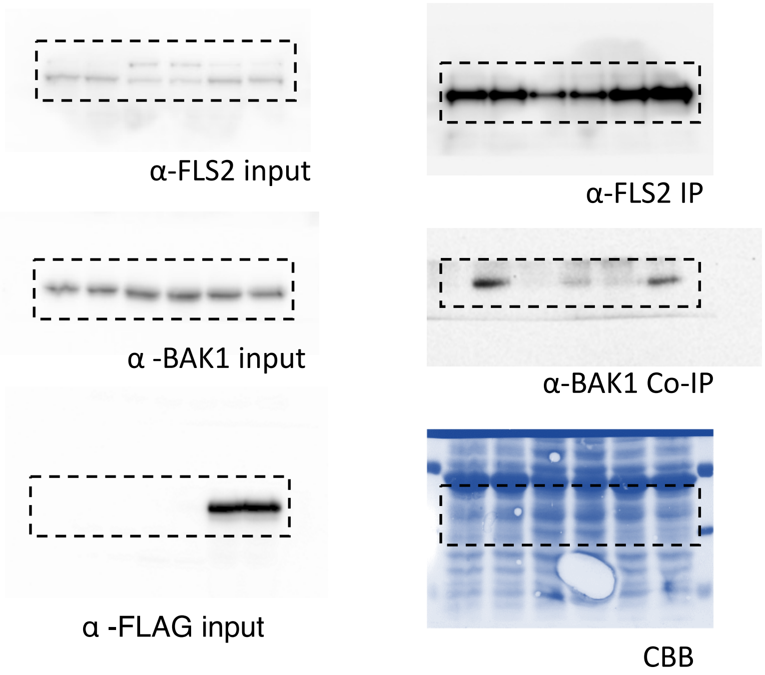

Source Data Figure 4 – supplement figure 8C

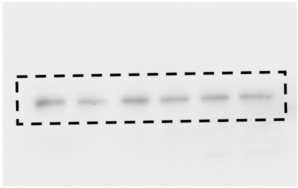

α-FLS2 input

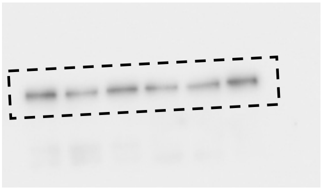

α-FLS2 IP

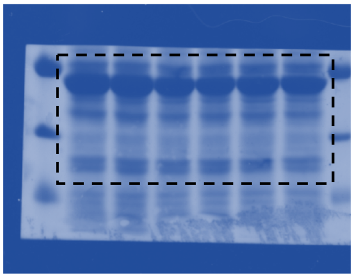

CBB

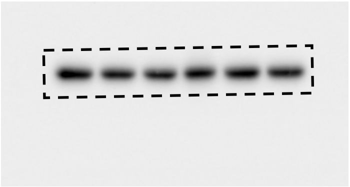

α -BAK1 input

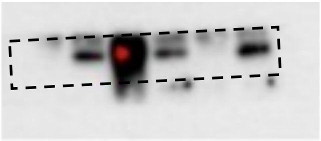

α-BAK1 Co-IP

Source Data Figure 4 – supplement figure 8D

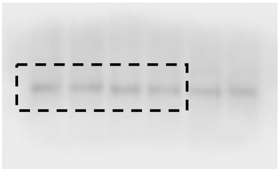

α-FLS2 input

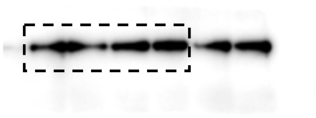

α-FLS2 IP

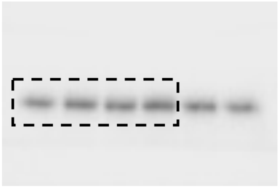

α -BAK1 input

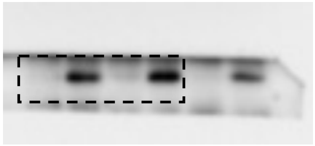

α-BAK1 Co-IP

Source Data Figure 4 – supplement figure 8G

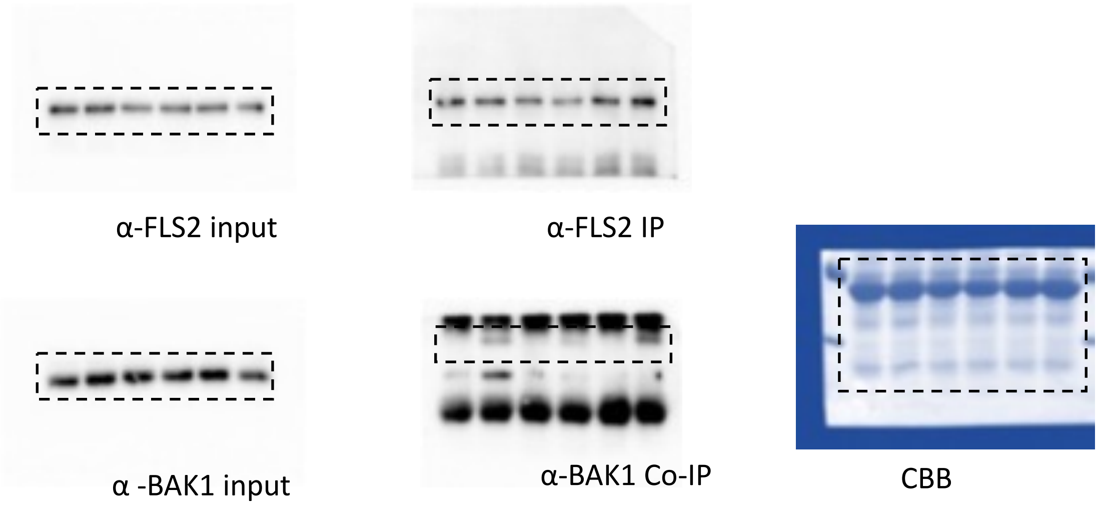

Source Data Figure 4 – supplement figure 8H

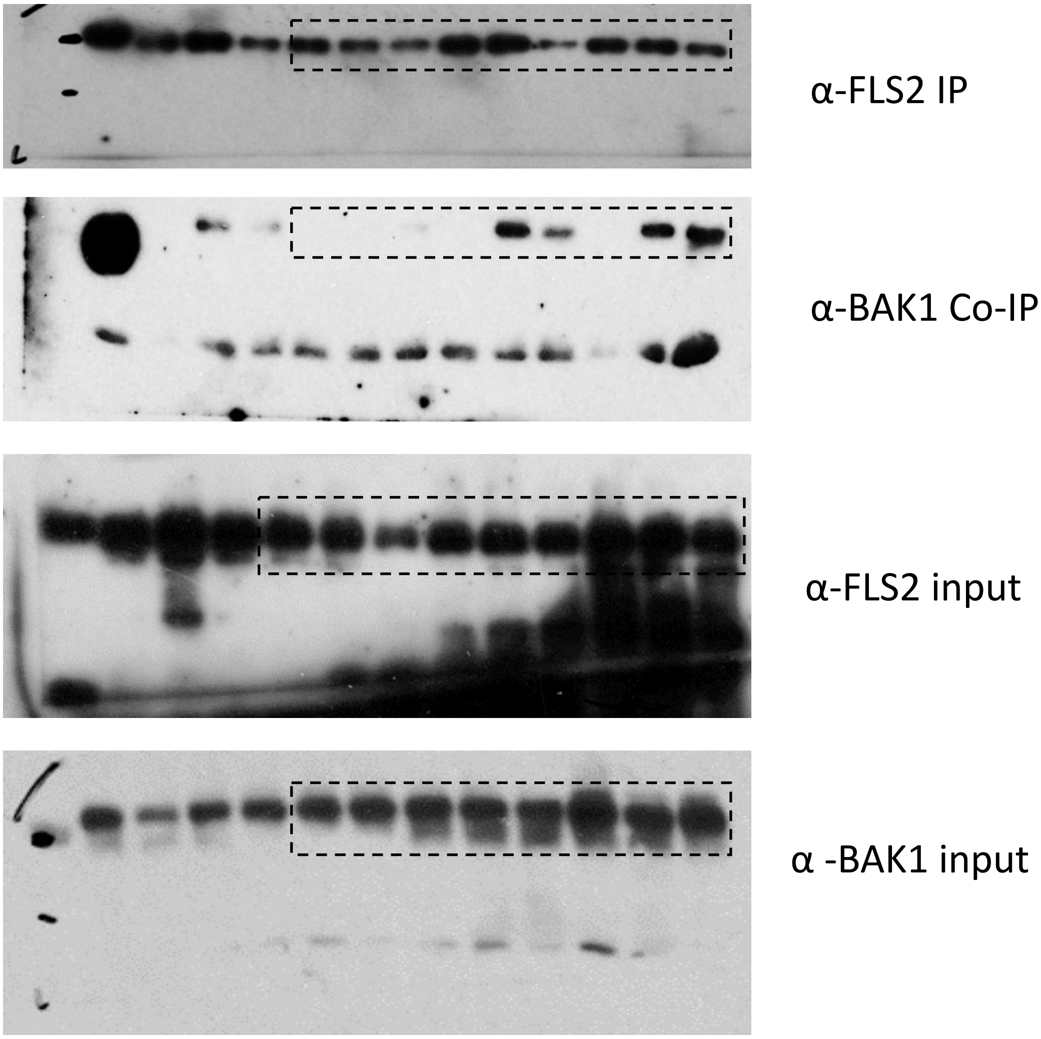

Source Data Figure 4 – supplement figure 8I

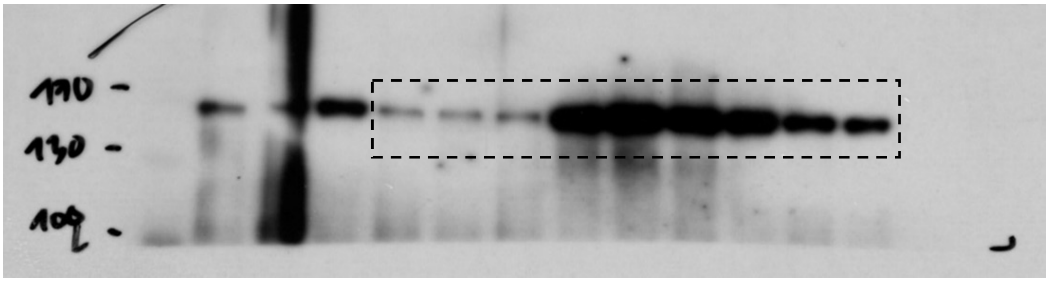

α-FLS2 IP

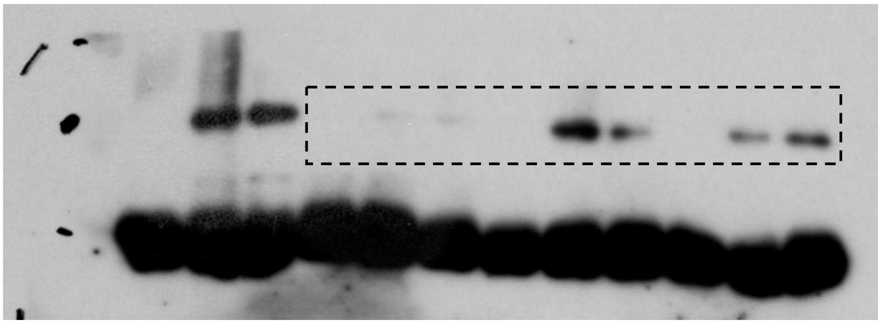

α-BAK1 Co-IP

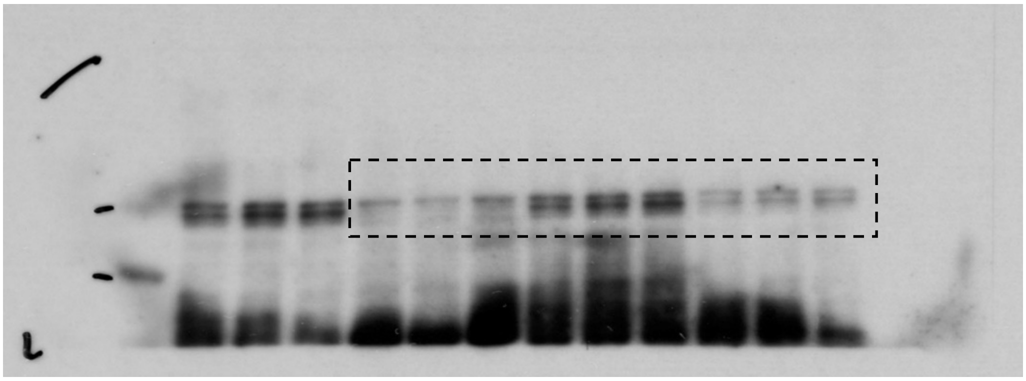

α-FLS2 input

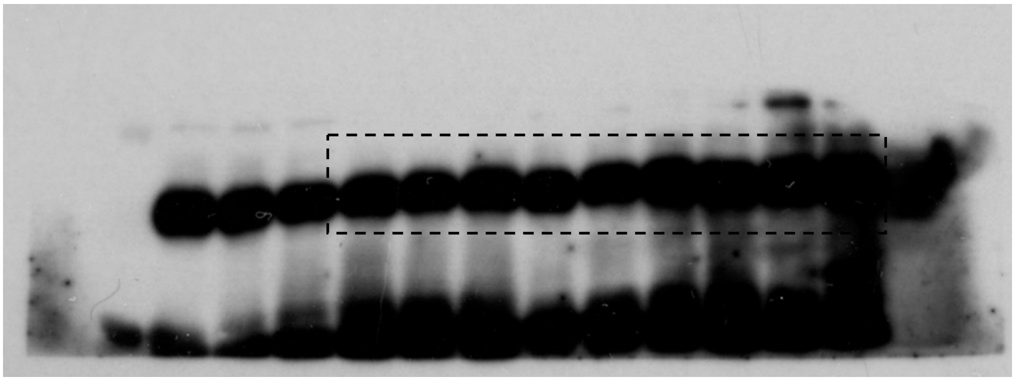

α -BAK1 input
