# Supplementary figures and images for: Regulation of immune receptor kinase plasma membrane nanoscale organization by a plant peptide hormone and its receptors (part 2 of 2)
Source: eLife. 2022 Jan 6;11:e74162. doi: 10.7554/eLife.74162 (PMC8791635; doi:10.7554/eLife.74162)

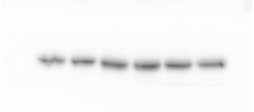

Supplement: Source data 1. [file elife-74162-supp1.zip › Figure 4 - supplement figure 8F/Input_BAK1.png]

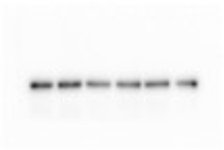

Supplement: Source data 1. [file elife-74162-supp1.zip › Figure 4 - supplement figure 8G/Input_FLS2.png]

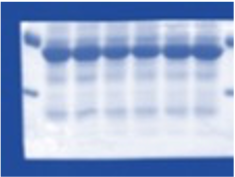

Supplement: Source data 1. [file elife-74162-supp1.zip › Figure 4 - supplement figure 8G/CBB.png]

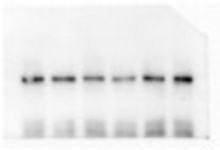

Supplement: Source data 1. [file elife-74162-supp1.zip › Figure 4 - supplement figure 8G/IP_FLS2.png]

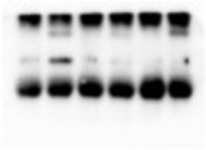

Supplement: Source data 1. [file elife-74162-supp1.zip › Figure 4 - supplement figure 8G/IP_BAK1.png]

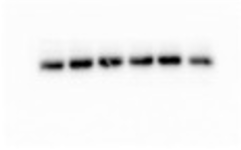

Supplement: Source data 1. [file elife-74162-supp1.zip › Figure 4 - supplement figure 8G/Input_BAK1.png]

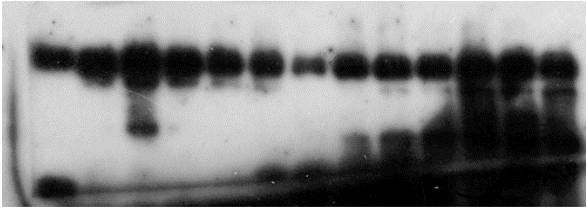

Supplement: Source data 1. [file elife-74162-supp1.zip › Figure 4 - supplement figure 8H/Input_FLS2.png]

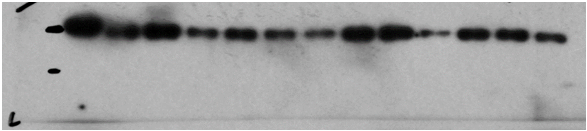

Supplement: Source data 1. [file elife-74162-supp1.zip › Figure 4 - supplement figure 8H/IP_FLS2.png]

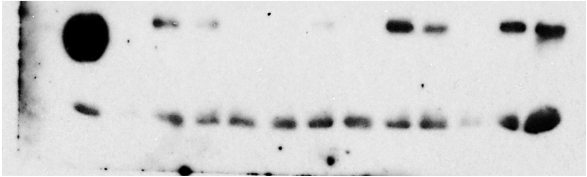

Supplement: Source data 1. [file elife-74162-supp1.zip › Figure 4 - supplement figure 8H/IP_BAK1.png]

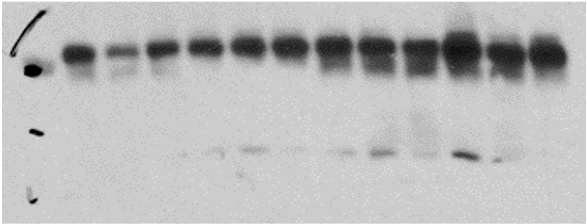

Supplement: Source data 1. [file elife-74162-supp1.zip › Figure 4 - supplement figure 8H/Input_BAK1.png]
